# GC-MS metabolite profiling of *Pseudocercospora fijiensis* isolates resistant to thiabendazole

**DOI:** 10.1371/journal.pone.0313915

**Published:** 2024-11-21

**Authors:** María Gabriela Maridueña-Zavala, Pablo Antonio Chong-Aguirre, Andrea Freire-Peñaherrera, Arturo Moreno, José Ignacio Reyes-De-Corcuera, María Isabel Jiménez-Feijoo, Juan Manuel Cevallos-Cevallos

**Affiliations:** 1 Centro de Investigaciones Biotecnológicas del Ecuador, Escuela Superior Politécnica del Litoral, ESPOL, Campus Gustavo Galindo, Guayaquil, Ecuador; 2 Facultad de Ciencias de la Vida, Escuela Superior Politécnica del Litoral, ESPOL, Campus Gustavo Galindo, Guayaquil, Ecuador; 3 Department of Agricultural and Biological Engineering, University of Florida, Gainesville, Florida, United States of America; Leibniz-Institut fur Naturstoff-Forschung und Infektionsbiologie eV Hans-Knoll-Institut, GERMANY

## Abstract

Black Sigatoka is the most widespread banana disease worldwide. It is caused by *Pseudocercospora fijiensis*, a fungal pathogen known for developing resistance to fungicides such as thiabendazole. Despite the increasing costs associated with the use of chemicals to control this disease, the pathogen’s mechanisms for fungicide resistance are not fully understood. The metabolite profiles of *P*. *fijiensis* isolates with different levels of resistance to thiabendazole were characterized by GC-MS. A total of 33 isolates were obtained from symptomatic banana plants and the sensitivity of each isolate to thiabendazole was assessed at 0, 1, 10, 100, 1000, and 10000 μg.mL^-1^. Then, the metabolite profile of each isolate was assessed using GC-MS. Metabolites such as hexadecanoic acid, tetradecanoic acid, octadecadienoic acid and octadecanoic acid were significantly over-accumulated in the presence of thiabendazole at 10 μg.mL^-1^. Phosphoric acid, L-proline, and D-allose increased in concentration with time in the presence of 100 μg.mL^-1^ of thiabendazole, and mannonic acid, 1-hexadecanol, D-sorbitol and tetracosanoic acid were only detected in the presence of the fungicide. Metabolic pathways including that of fructose, mannose metabolism, the biosynthesis of unsaturated fatty acids, and ABC transporters were upregulated in resistant isolates. Our findings show an increment of tetracosanoic (myristic) acid suggesting a possible β-tubulin-compensation mechanism in resistant isolates. The presence of myristic acid promoted the generation of diacylglycerol kinase δ which facilitated the production of β-tubulin in other studies. Additionally, important changes in the metabolite profiles were observed as soon as six hours after exposure to the fungicide showing an early response of the pathogen. To the best of our knowledge, this is the first report that describes the changes in the metabolite profile of *P*. *fijiensis* resistant to thiabendazole when exposed to the fungicide.

## Introduction

The production of banana (*Musa* sp.) is considered one of the most important economic, nutritional and social activities in many tropical countries. Ecuador is the top banana exporter and one of the biggest producers of this commodity, with a total production of 6.6 million tons a year which represents almost 6% of the bananas produced in the world [1]. Bananas in Ecuador are cultivated in Los Rios (56,046 ha), El Oro (49,130 ha), Guayas (50,719 ha) and other provinces (15.002 ha) with a total of 170,897 ha [2]. However, banana production is affected by black leaf streak disease (BLSD), commonly known as black Sigatoka disease, caused by the ascomycete *Pseudocercospora fijiensis*. BLSD is characterized by a gradual deterioration of the leaves, affecting their photosynthetic action and growth prior the development of a strong necrosis [3] and premature ripening of the fruit, causing a decrease in fruit quality and yield [4]. Due to its sexual reproduction and short life cycle, *P*. *fijiensis* exhibits a high level of genetic diversity, which results in numerous generations per year with a high rate of genetic recombination [5].

Fungicide treatments are an important component of an integrated management of plant diseases. Particularly, benomyl [6], propiconazole [7] and azoxystrobin fungicides have been widely used for BLSD control [8, 9]. However, development of fungicide resistance has become a major limiting factor affecting the efficacy and lifetime of fungicides [10]. Therefore, rotation programs with increasing doses of different classes of fungicides with different activities are usually implemented as a response to the fungicide resistance development in *P*. *fijiensis*. Chemical control of BLSD can represent around 40 to 60% of the total cost of banana production [3]. Additionally, the excessive use of fungicides has caused environmental damage, impacted the health of exposed human populations, and induced the pathogen’s loss of sensitivity to various systemic fungicides [11]. However, the fungicide resistance mechanisms are still not fully understood.

Thiabendazole together with benomyl belongs to the methyl benzimidazole carbamates fungicide group (MBCs). Both fungicides were among the first modern site-specific fungicides used for disease control in bananas [12–14]. Thiabendazole has been regarded as a highly systemic and curative fungicide [13, 14] able to control pathogens before infection, in early stages and in already colonized tissue. Thiabendazole mode of action has been associated with the inhibition of the mitochondrial enzyme fumarate reductase, disrupting the citric acid cycle, mitochondrial respiration, and subsequent ATP production leading to cell death [15, 16]. Additionally, in the case of *P*. *fijiensis* and many other fungi; thiabendazole inhibits microtubule polymerization by binding to β-tubulin [10, 16, 17]. However, *P*. *fijiensis* resistance to the MBCs fungicide group was first detected as early as 1976 [14] making MBCs fungicides currently obsolete and thiabendazole is no longer used for the control of *P*. *fijiensis* (FRAC 2018). Still, the *P*. *fijiensis* resistance mechanisms against thiabendazole are not fully understood.

Acquired fungicide resistance in *P*. *fijiensis* has been assessed by molecular markers, expressed sequence tags (ESTs) [18], cloning and sequencing of target gene [19] identification of resistance locus, segregation pattern of sensitive and resistant crosses [20], affinity of fungicide to target enzyme, transport and degradation of fungicide, among others [9]. Results suggest that the β-tubulin specific substitution E198A in *P*. *fijiensis* may confer resistance to the fungicide in some isolates [10, 24, 31]. However, molecular methods alone have not been able to fully characterize fungicide-resistance mechanisms in plant pathogens, and other tools such as metabolomics have been proposed [21].

Metabolomics have been used to assess the changes in the metabolite profile of living organisms as a response to external factors. Metabolomic techniques based on gas chromatography coupled to mass spectrometry (GC-MS) have been applied for the characterization of physiological changes in fungi [21–23] due to the high sensitivity and reproducibility of GC-MS instruments with low running costs. GC-MS specializes in small molecular weight metabolites which are typically produced by microorganisms such as plant pathogens [21]. The use of GC-MS suggested that the fungicide (metalaxyl) resistance mechanisms in *Phytophthora infesntans* were associated with the regulation of the pathogen’s membrane fluidity by fatty acid biosynthesis and glycerophospholipid metabolism [21]. However, the metabolomic assessment of the fungicide resistance in *P*. *fijiensis* has not been reported. This research aimed to elucidate the mechanisms of resistance to thiabendazole in *P*. *fijiensis* by characterizing the GC-MS metabolite profile of isolates with different levels of resistance to the fungicide.

## Materials and methods

### *Pseudocercospora fijiensis* isolates

A total of 33 monosporic isolates of *P*. *fijiensis* from Guayas (13 isolates), El Oro (9), Esmeraldas (5) and Los Rios (6) provinces were obtained from the Culture Collection of Microorganism from CIBE-ESPOL (CCM-CIBE) in Guayaquil-Ecuador. The strains were propagated using potato dextrose agar (PDA) and incubated at 28 °C under dark conditions for 45 days to promote mycelial growth and conidia production [24]. Then, DNA was extracted from each isolate and Sanger sequencing of the ITS (Internal transcribed spacer) and LSU (Large subunit) regions was carried out to confirm the pathogen’s identity. Briefly, DNA extraction was performed using LinkTM Genomic DNA Mini-Kit (Thermo Fisher Scientific Inc.) followed with PCR amplifications with primers ITS1 (5’-TCCGTAGGTGAACCTGCGG-3’) and ITS4 (5’-TCCTCCGCTTATTGATATGC-3’) [25]. The LSU region was also partially amplified using the primers LROR (5’-ACCCGCTGAACTTAAGC-3’) and LR3 (5’-CCGTGTTTCAAGACGGG-3’). Polymerase chain reaction was performed using a final reaction mix of 25 μL containing: DNA, dNTP (final concentration = 0.20 mM), primers, water, and GoTaq^®^ Green Master Mix (Promega, Madison, USA), according to the manufactured procedures. Thermocyclers amplification was done at 94 °C for 1 min, followed by 30 cycles of 94 °C for 1 min, 52 °C for 30 s and then a final step at 72 °C for 10 min. The fragments generated were separated in a 1.5% agarose gel by an electrophoresis run in 1X TAE (Tris base, boric acid and EDTA 0.5 M, pH 8.0) at 120 V during 20 min using 5 μL of PCR product with 1 μL of loading dye (Promega, USA). The gel images were analyzed using the program Imager Gel Doc XR (Bio-Rad, Philadelphia, California, USA) and the size was estimated using the 100 bp Low DNA Mass^™^ Ladder (Invitrogen^™^). A spectrophotometer model NanoDrop 2000 from Thermo Fisher Scientific Inc. (Wilmington, DE, USA), was used to estimate the DNA concentrations (260 nm) and quality (260/280 nm and 260/230 nm) before sending the PCR product to a service lab for Sanger sequencing.

### Sensitivity test

To determine the sensitivity of 33 isolates to thiabendazole, the minimum inhibitory concentration (MIC) method was used. Briefly, a 100,000 μg.mL^-1^ stock solution of the fungicide thiabendazole (Sigma, USA) was prepared using pure dimethyl sulfoxide (DMSO) from MP Biomedicals (Santa Ana, CA, USA), followed by filter sterilization using 0.2 μm syringe filters. The stock solution was then mixed with PDA to obtain Petri dishes with 0, 1, 10, 100, 1000, and 10000 μg.mL^-1^ of thiabendazole in PDA. Then, a small mycelium fragment from each isolate was cut using a scalpel and inoculated onto Petri dishes containing PDA amended with the different concentrations of thiabendazole and incubated for 34 days at 28 °C prior to metabolomic analysis by GC-MS. All analysis were done in triplicate and the MIC values were determined at the concentrations that completely prevented visible growth of the tested isolate [26]. In this study, isolates with MIC of 10 μg.mL^-1^ or lower were considered sensitive to thiabendazole, whereas isolates with MIC of 100 μg.mL^-1^ or higher were regarded as resistant to the fungicide [27].

### Thiabendazole effect on the metabolite profile of *P*. *fijiensis*

The effect of thiabendazole on the metabolite profile of resistant isolates of *P*. *fijiesis* was assessed in two experiments.

The first experiment was designed to evaluate the effect of thiabendazole concentration on the metabolite profile of resistant isolates of *P*. *fijiesis*. In this experiment, selected resistant isolates of the pathogen were inoculated onto PDA amended with 0, 10 and 100 μg.mL^-1^ of thiabendazole and incubated at 28 ºC for 28 days. Then, the metabolite profile of each isolate was assessed by GC-MS.

The second experiment was designed to evaluate the effect of thiabendazole exposure time on the metabolite profile of resistant isolates of *P*. *fijiesis*. To provide a deeper insight into resistance mechanisms, the effect of the fungicide at 6, 24 and 48 h after inoculation was assessed on resistant isolates. To achive this, mycelial discs of each isolate showing a MIC of 10,000 μg.mL^-1^ were plated onto PDA and incubated for 15 days at 28 °C. Mycelial discs of each isolate were then transferred into 100-mL Erlenmeyer flasks containing 45 mL of potato dextrose broth (PDB). Liquid media (PDB) was used for this experiment to allow the pathogen to produce sufficient biomass for analysis prior to adding the fungicide. Flasks were closed using a cotton cap and incubated at 28 ºC in a Excella E24 Incubator Shaker Series (New Brunswick Scientific, Edison, NJ) at 120 RPM for 25 days. Then, thiabendazole was carefully added to each flask to a final concentration of 100 μg/mL and the incubation continued under the same conditions for two additional days. Mycelial samples were carefully recovered using sterile loops at 6, 24 and 48 h after fungicide addition and submitted to metabolomic analysis by GC-MS.

### Metabolomic analysis by gas chromatography-mass spectrometry

The metabolomic analysis by GC-MS was carried out as described by Maridueña et al, 2017 [21] with small modifications. Briefly, 400 mg of *P*. *fijiensis* mycelia from each experiment were transferred into 2-mL tubes containing 1.5 mL of methanol-chloroform-water (8:1:1) and incubated at 7 °C for 48 h. The suspensions were then centrifuged at 15,000 × g for 4 min using a microcentrifuge model 5424 from Eppendorf (Hamburg, Germany) and 600 μL of the supernatant was transferred to new tubes and incubated with the cap open in a water bath at 80 °C until completely dry. Sample derivatization was performed by adding 150 μL of N-methyl- N-butyldimethylsilyltrifluoroacetamide (MSTFA Thermo Scientific) to the dried samples and incubated in a water bath at 85 ºC with the cap closed for 90 min. Volumes of 1 μL of derivatized samples were splitlessly injected into a GC-MS (7890A / 5975C, Agilent Technologies, Santa Clara, CA) containing a HP5 column of 30 m length and 0.5 μm diameter and operating under the following conditions: The injector was at 240 ºC, the oven operated at 70 ºC for 1 min with a 7 ºC/min ramp with a final temperature of 310 ºC held for 5 min. Ultrapure helium was used as the carrier gas at 1 mL/min. The GC-MS interface was set to 280 °C and after 8 min of solvent delay the scan was recorded with a frequency of 4 s^−1^ in a total scan MS mode with 50–650 m/z detection range. Differentially accumulated metabolites (i.e. metabolites showing significant differences after data analysis) were putatively identified by matching their MS spectra to the compounds in both NIST 11 and Wiley 9 databases. Metabolite identity was then confirmed by comparing the linear retention index of each differentially accumulated metabolite with that of the pure standard using our internal database.

Quality of the GC-MS runs was assessed by running one selected extract every five runs and estimating the variations in retention time and peak areas. Maximum acceptable coefficient of variation was 30% for a given metabolite in QC runs [27].

### Data analysis

GC-MS data were aligned using MZmine 3.4 [28] and normalized to the total area. Principal component analysis (PCA) was carried out on XLSTAT (Addinsoft, Paris) using the overall metabolite profile [29].

Differentially accumulated metabolites were selected using t-test at the 0.05 significance level and the fold change of each metabolite after thiabendazole exposure was reported as the logarithm base 2 (Log2FC) using RStudio (version 4.3.2) [30]. Differentially accumulated metabolites were mapped into the KEGG Pathway database [31] set to *P*. *fijiensis* as the model organism to determine the potentially affected metabolic pathways. The Pearson coefficient was estimated to correlate the intensities of each metabolite with the thiabendazole concentrations or exposure time. Heatmaps were built in Heatmapper.ca [29] using the abundances of differentially accumulated metabolites and Volcano plots were built using the Log2FC as well as the p-values of all metabolites using the ggplot2 package in R. Compounds with -log10(p-value) > 1.3 and |Log2FC| > 0.6 were reported as the differentially regulated metabolites in the volcano plot [32].

## Results

The study of the metabolites profiles of *P*. *fijiensis* isolates susceptible and resistant to different fungicide doses allows to identify biochemical pathways involved in resistance. The aim of this study was to characterize metabolites from resistant and susceptible strains grown in PDA amended with 10, 100, 1000 and 10000 μg.mL^-1^ of thiabendazole. Then, selected resistant isolates were evaluated in liquid medium exposed to the fungicide for 6, 24 and 48 hours. Our study included 33 *P*. *fijiensis* strains isolated from different Ecuadorian provinces and conserved in the CCM-CIBE. Sequencing of the ITS and LSU region of the 33 isolates shared a 99% of similarity with *P*. *fijiensis* sequences from the NCBI data base (S1 Fig).

### Sensitivity of *P*. *fijiensis* to thiabendazole

Fungicide resistance was assessed by plating each isolate onto PDA amended with 4 different doses of thiabendazole. Results showed that 10 isolates were sensitive to fungicide concentrations below 10 μg.mL^-1^ or less. The remaining 23 isolates were classified as resistant as they were able to grow at thiabendazole concentrations below 100 μg.mL^-1^ (12 isolates), 1,000 μg.mL^-1^ (4 isolates) and 10,000 μg.mL^-1^ (7 isolates) (Table 1).

**Table 1:** Growth of *P*.*fijiensis* isolates on PDA amended with 10, 100, 1,000 and 10,000 μg.mL^-1^ of thiabendazole during 28 days.

Province	Isolate code	Growth on thiabendazole concentration*			
		10 μg.mL^-1^	100 μg.mL^-1^	1,000 μg.mL^-1^	10,000 μg.mL^-1^
*El Oro*	Osa 26	x	MIC		
*El Oro*	Ossr 95	x	MIC		
*El Oro*	Ons51	x	MIC		
*El Oro*	Ocm 37	x	x	MIC	
*El Oro*	Ossr 151	x	x	MIC	
*El Oro*	Ossr 100	x	x	MIC	
*El Oro*	Ossr 84	x	x	x	MIC
*El Oro*	Osa 6	x	x	x	MIC
*El Oro*	Onm41	MIC			
*Esmeraldas*	Env1	MIC			
*Esmeraldas*	En1	MIC			
*Esmeraldas*	En3	MIC			
*Esmeraldas*	Enb25	MIC			
*Esmeraldas*	Ecu12	x	x	MIC	
*Guayas*	Salv1#7	MIC			
*Guayas*	#1	MIC			
*Guayas*	#3	MIC			
*Guayas*	#4	MIC			
*Guayas*	Gna 12	x	MIC		
*Guayas*	Gnm5	x	MIC		
*Guayas*	G54	x	MIC		
*Guayas*	#2	x	MIC		
*Guayas*	Gnm1	x	x	x	MIC
*Guayas*	Gs 89	x	x	x	MIC
*Guayas*	Gsa 16	x	x	x	MIC
*Guayas*	Gsn84	x	x	x	MIC
*Guayas*	Gsa1	x	x	x	MIC
*Los Ríos*	Salv2#6	x	MIC		
*Los Ríos*	Salv2#8	x	MIC		
*Los Ríos*	Convc#5	x	MIC		
*Los Ríos*	Convc#6	x	MIC		
*Los Ríos*	Convc#7	x	MIC		
*Los Ríos*	Convc#8	MIC			

* x = Normal growth; MIC = Minimum inhibitory concentration (no growth at this or higher fungicide concentrations)

### Effect of thiabendazole concentration in the metabolite profile of *P*. *fijiensis*

The metabolite profile of sensitive (MIC = 10 μg.mL^-1^) and resistant isolates (MIC ≥ 100 μg.mL^-1^) was assessed using PDA at 0, 10 and 100 μg.mL^-1^ thiabendazole. A total of 69 metabolites were detected across all isolates of *P*. *fijiensis* and the overall metabolite profile of all isolates was used to perform PCA. However, no separation was evidenced between control and resistant groups in the PCA score plot (S2 Fig).

On the other hand, univariate analysis using T-test showed that a total of 22 metabolites were differentially (over- or under-) accumulated (p < 0.05) in the pathogen when exposed to thiabendazole concentrations of 10 μg.mL^-1^ and 100 μg.mL^-1^ (Table 2). The intensities of all metabolites—except myristic and octadecanoic acids—showed a negative correlation with the fungicide concentration with potential effect on various metabolic pathways including fructose and mannose metabolism, galactose metabolism, ABC transporters, biosynthesis of unsaturated fatty acids, arachidonic acid metabolism, Fatty acid elongation, arginine and proline metabolism, and biosynthesis of amino acids (Table 2).

**Table 2:** Differentially accumulated (p < 0.05) metabolites in *P*. *fijiensis* exposed to 10 and 100 μg.mL^-1^ thiabendazole in PDA after the 28 days of mycelium growth of the fungi.

*Compound* *	*LOG*_*2*_ *FC 10*	*LOG*_*2*_ *FC 100*	*Pearson correlation coefficient* **	*Potential metabolic pathway*
Eicosanoic acid	0.529	NDF	-0,92	
Mannonic acid	NDC	NDC, NDF	-0,42	
9,12-Octadecadienoic acid	2.943	NDF	-0,52	Together with tetradecanoic acid are important for cell membrane integrity in bacteria resistance to bile
Octadecane	-1.319	NDF	-0,85	
Allyl tert-butyldimethylsilyl phthalate	1.605	NDF	-0,69	
1-Hexadecanol	NDC	NDC, NDF	-0,42	Fatty acid degradation
D-sorbitol	NDC	NDC, NDF	-0,42	Fructose and mannose metabolism, galactose metabolism, ABC transporters
Octadecanoic acid	1.707	0,623	1,00	Biosynthesis of unsaturated fatty acids, Fatty acid biosynthesis
d-ribo-hexitol,3-deoxy	0.620	NDF	-0,90	
Tetradecanoic acid (Myristic acid)	3.005	-0.206	0,92	Myristic acid treatment increased the protein level of β-tubulin, which constitutes microtubules in humans [33]
Oleic acid	1.909	NDF	-0,64	Biosynthesis of unsaturated fatty acids, Arachidonic acid metabolism, Metabolic pathways
Phosporic acid	-1.038	-2.884	-0,52	
6-Octadecenoic acid	2.688	NDF	-0,55	
Heptadecanoic acid	2.884	NDF	-0,53	
Docosonoic acid	4.306	NDF	-0,46	
D-(+)-Trehalose	2.206	NDF	-0,60	
Palmitic acid (Hexadecanoic acid)	1.337	NDF	-0,74	Biosynthesis of unsaturated fatty acids, fatty acid biosynthesis, fatty acid degradation, fatty acid elongation, fatty acid metabolism
L-Proline	-1.523	NDF	-0,82	Arginine and proline metabolism, ABC transporters, carbapenem biosynthesis, aminoacyl-tRNA biosynthesis, biosynthesis of secondary metabolites, biosynthesis aminoacids, biosynthesis of antibiotics
Myo-Inositol	3.101	NDF	-0,51	Glycosylphosphatidylinositol (GPI)-anchor biosynthesis, glycosylphosphatidylinositol (GPI)-anchor biosynthesis, inositol phosphate metabolism, phosphatidylinositol signaling system, endocytosis, galactose metabolism, autophagy—other—*Pseudocercospora fijiensis*, autophagy—yeast—*Pseudocercospora fijiensi*s, Phagosome, ascorbate and aldarate metabolism, glycerophospholipid metabolism, AGE-RAGE signaling pathway in diabetic complications
1-Nonadecene	-3.226	NDF	-0,65	
Pentadecane, 8-hexyl- / Eicosane, 2-Methyl	-2.364	-4.352	-0,74	
Tetracosanoic acid	NDC	NDC, NDF	-0,42	Biosynthesis of unsaturated fatty acids

*NDC = Not detected in control samples.

NDF = Not detected in samples exposed to the fungicide at concentration indicated in the column.

** Correlation between metabolite intensity and thiabendazole concentration.

**Fig 1:**
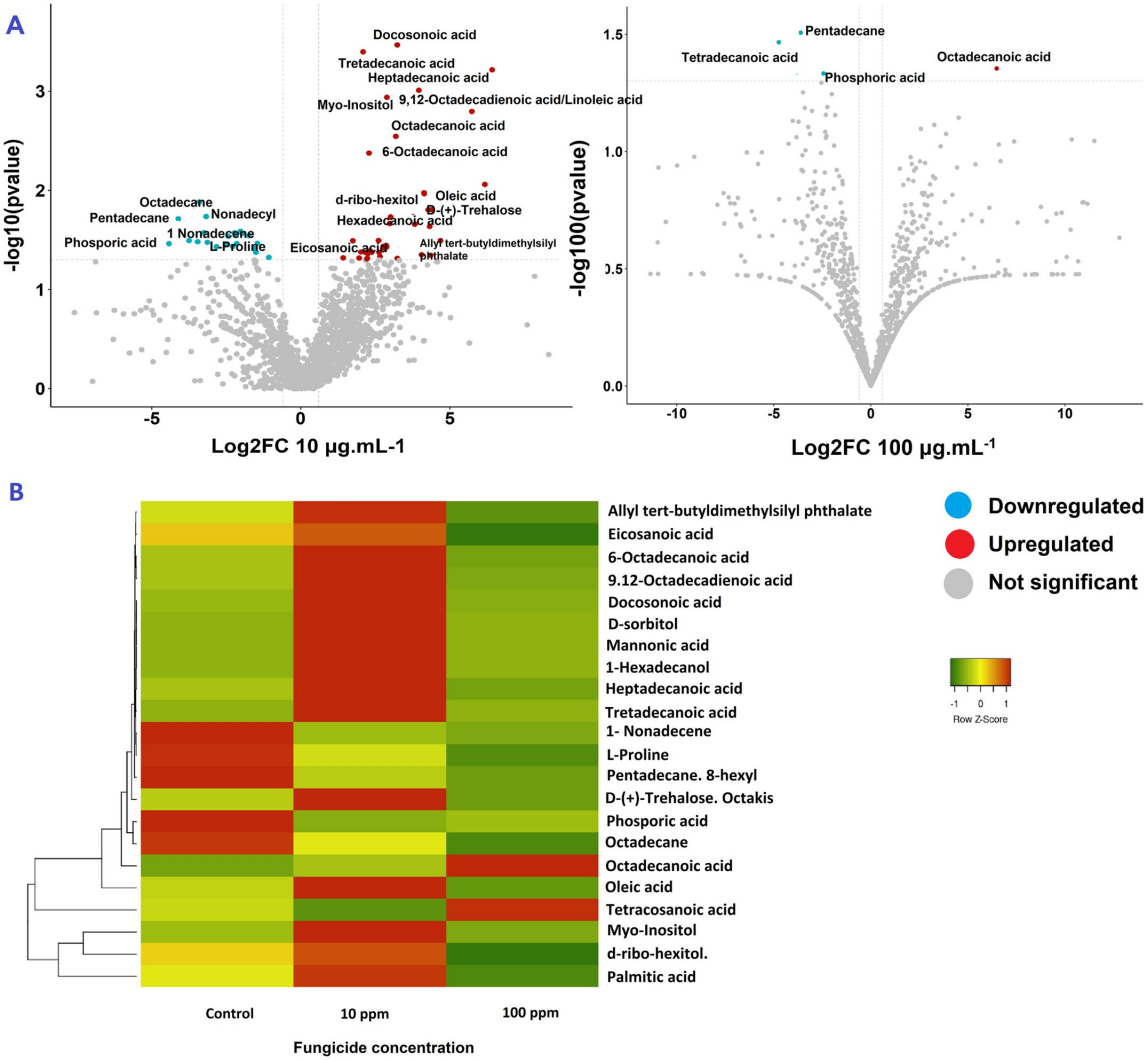
Effect of fungicide concentrations in the metabolite profile of *P*. *fijiensis* resistant to thiabendazole. (A) Volcano plot of the metabolites found and 10 μg.mL^-1^ and 100 μg.mL^-1^. (B) Heatmap of differentially accumulated metabolites of *Pseudocercospora fijiensis* obtained in samples grown on PDA with 0 (control), 10 and 100 μg. mL^-1^ of thiabendazole after 28 days.

The volcano plot showed that the only over-accumulated (upregulated) metabolite at both 10 μg.mL^-1^ and 100 μg.mL^-1^ was octadecanoic acid; whereas the levels of phosporic acid, and pentadecane, 8-hexyl- / eicosane, 2-methyl were significantly suppressed (downregulated) by the fungicide at both concentrations (Fig 1).

Several metabolites, including mannonic acid, 1-hexadecanol, D-sorbitol, and tetracosanoic acid were only detected in samples exposed to 10 μg.mL^-1^. Eicosanoic acid; 9,12-octadecadienoic acid, octadecane, allyl tert-butyldimethylsilyl phthalate, d-ribo-hexitol,3-deoxy, oleic acid, 6-octadecanoic acid, heptadecanoic acid, docosonoic acid, D-(+)-trehalose, palmitic acid, L-proline, myo-inositol, and 1-nonadecene were present in isolates exposed to 10 μg.mL^-1^ but were not detected in resistant isolates exposed to 100 μg.mL^-1^ (Table 2).

### Exposure time effect on the metabolite profile of *P*. *fijiensis*

**Fig 2:**
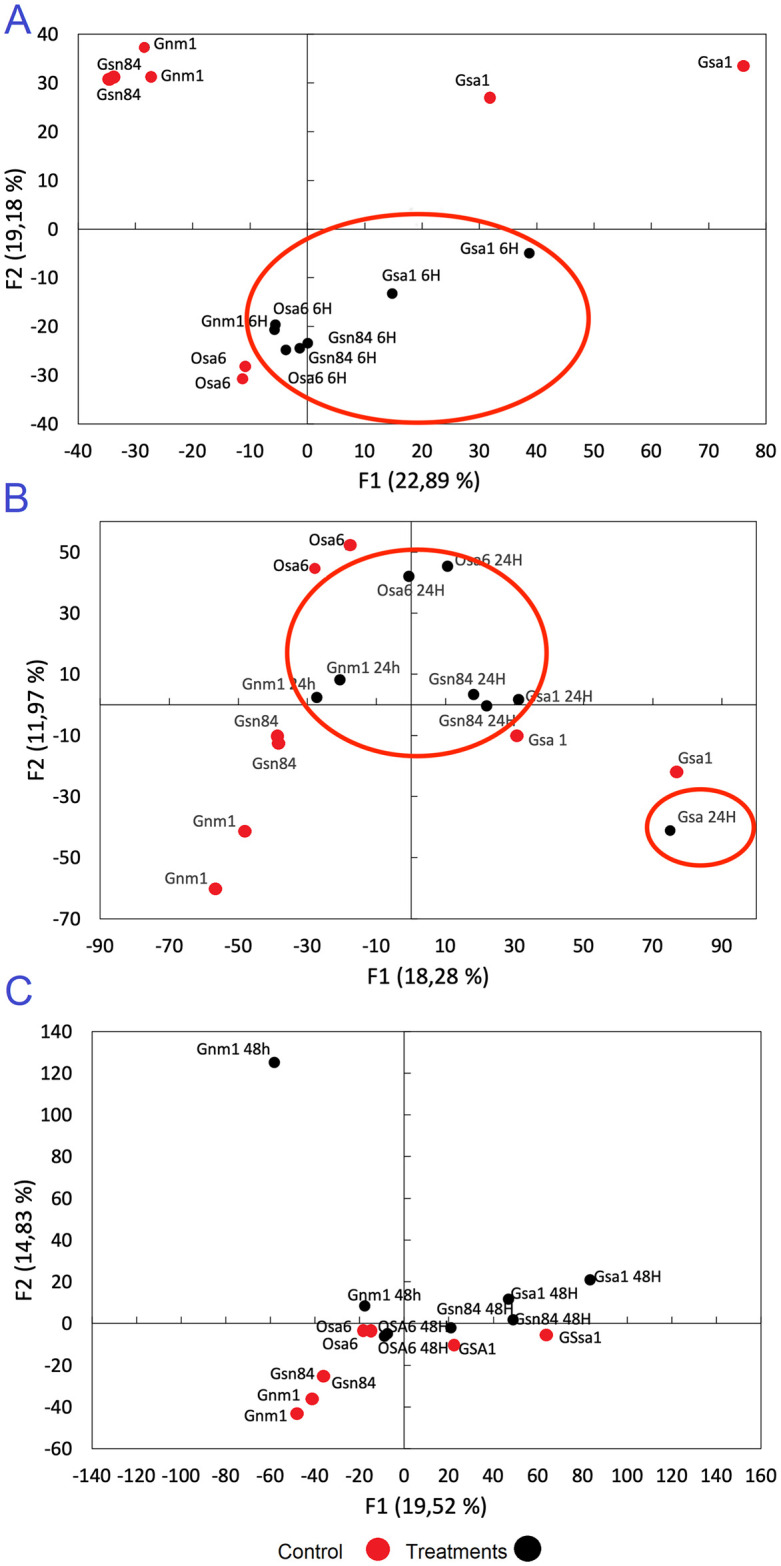
Principal component analysis of *P*. *fijiensis* metabolites exposed to thiabendazole at different timepoints. PCA of the metabolite profile of *P*. *fijiensis* grown during 6 (A), 24 (B) and 48 (C) hours in PDB amended with 100μg.mL^-1^ thiabendazole. Isolates codes are from Table 1. The suffixes 6H, 24H and 48H represent the fungicide exposure time and are represented by the black dots. No suffix represent isolates grown with no fungicide (red dots). Significant groupings of fungicide-exposed isolates—when observed—are highlighted in red circles.

To understand the changes that occurred in the pathogen’s metabolite profile during the initial hours of contact with the fungicide, four resistant isolates (Gsa1, Osa6, Gsn84, and Gnm1 from Table 1) were evaluated at 6, 24 and 48 h after exposure to 100 μg.mL^-1^ thiabendazole. This concentration of the fungicide was selected for this experiment as the most representative MIC of the isolates evaluated. A total of 69 metabolites were detected across all isolates of *P*. *fijiensis* and the overall metabolite profile of all isolates was used to perform PCA (Fig 2). The PCA revealed that samples were significatively grouped according the fungicide exposure time. After 6 and 24 hours of fungicide exposure, control samples (samples exposed to 0 μg.mL^-1^ of the fungicide) grouped separately from the samples exposed to 100 μg.mL^-1^ of thiabendazole, except for one isolate from the Guayas province (Fig 2A and 2B). However, separation of samples groups was not significant after 48 hours of exposure (Fig 2C). The loading plot revealed that the metabolites that contributed the most to the grouping observed at 6 and 24 hours of fungicide exposure were the same as the differentially accumulated metabolites selected by t-test (Table 3).

**Table 3:** Differentially accumulated (p < 0.05) metabolites in *P*. *fijiensis* after 6 h, 24 h and 48 h exposure to 100 μg.mL^-1^ of thiabendazole in PDB.

*Compound* *	*LOG*_*2*_ *FC 6H*	*LOG*_*2*_ *FC 24H*	*LOG*_*2*_ *FC 48H*	*Pearson correlation coefficient* **	*Potential metabolic pathways*
Phosphoric acid	1,310	1,441	1,585	0,56	ABC transporters, selenocompound metabolism, metabolic pathways
L-Proline, 5-oxo-	NDF	3,337	-7,338	-0,59	Arginine and proline metabolism, ABC transporters, carbapenem biosynthesis, aminoacyl-tRNA biosynthesis, biosynthesis of secondary metabolites, biosynthesis of antibiotics, biosynthesis of amino acids
Gulonic acid, 1,4-lactone	NDF	NDF	1,655	0,82	
Dulcitol	3.980	5,668	2,946	-0,34	
Arabino-hexaric acid, 3-deoxy-2,4,5-tris	NDF	2,315	-1,796	-0,44	
D-Ribo-hexonic acid, 3-deoxy	-2,728	-1,868	-3,803	-0,83	
D-(-)-Fructofuranose	-8,630	-3,052	-3,274	-0,53	
D-Sorbitol	NDF	-2,786	-3,789	-0,53	Fructose and mannose metabolism, galactose metabolism, ABC transporters
alfa-D-glucopyranose	-5,573	0,016	-2,246	-0,38	Metabolic pathways, biosynthesis of secondary metabolites, biosynthesis of antibiotics, Inositol phosphate metabolism, carbon metabolism.
D-Allose	2,780	1,894	2,685	-0,41	Fructose and mannose metabolism, ABC transporters
D-(+)- Galacturonic acid	-0,642	3,579	3,793	0,98	
Octadecanoic acid	4,470	2,166	-3,527	-0,42	Biosynthesis of unsaturated fatty acids, fatty acid biosynthesis
N-Acetyl glucosamine methoxime	NDF	NDF	NDF	-0,60	Other types of O-glycan biosynthesis, mannose type O-glycan biosynthesis, amino sugar and nucleotide sugar metabolism, ABC transporters, glycosylphosphatidylinositol (GPI)-anchor biosynthesis
D-Psicose	NDF	NDF	-3,047	-0,51	
B-d-Fructopyranose	-4,241	-4,219	-2,615	-0,61	
D-(+)-Cellobiose	NDF	-1,010	2,122	-0,12	
4-O-B-Galactopyranosyl-D-mannopyranose	-5,769	-1,502	-3,849	-0,55	
2-alfa-mannobiose	NDF	-1,010	NDF	-0,29	
Glycerol	NDC	NDC, NDF	NDC, NDF	-0,42	
Thiabendazole	NDC	NDC	NDC	0,02	
1,11-Undecanedioic acid	NDC, NDF	NDC	NDC, NDF	0,14	
Butanedioic acid (Succinic acid)	NDF	-2,239	1,898	0,13	Citric acid cycle, mitochondrial respiration, and ATP production
Propanoic acid	NDC, NDF	NDC, NDF	NDC	0,88	Phenylalanine metabolism
d-Ribose	NDF	NDF	1,184	0,67	
Benzoic acid	NDF	2,421	2,660	0,87	
Inositol	NDF	1,940	3,157	0,81	Glycosylphosphatidylinositol (GPI)-anchor biosynthesis, inositol phosphate metabolism, phosphatidylinositol signaling system, endocytosis,

*NDC = Not detected in control samples.

NDF = Not detected in samples exposed to the fungicide at concentration indicated in the column.

** Correlation between metabolite intensity and thiabendazole exposure time.

Univariate t-test revealed that a total of 26 metabolites were differentially accumulated in samples exposed to thiabendazole for 6, 24 and 48 hours. The intensities of all metabolites—except phosphoric, gluconic, galacturonic, 1,11 undecanoic, succinic, propanoic and benzoic acids, as well as intracellular thiabendazole, ribose and inositol—showed a negative correlation with the fungicide exposure time. Similar to what was observed after the fungicide concentration evaluation, metabolite mapping to the KEGG database showed that fructose and mannose metabolism, galactose metabolism, ABC transporters, biosynthesis of unsaturated fatty acids, arachidonic acid metabolism, Fatty acid elongation, arginine and proline metabolism, and biosynthesis of amino acids were the main pathways potentially affected by the fungicide in resistant isolates (Table 3).

**Fig 3:**
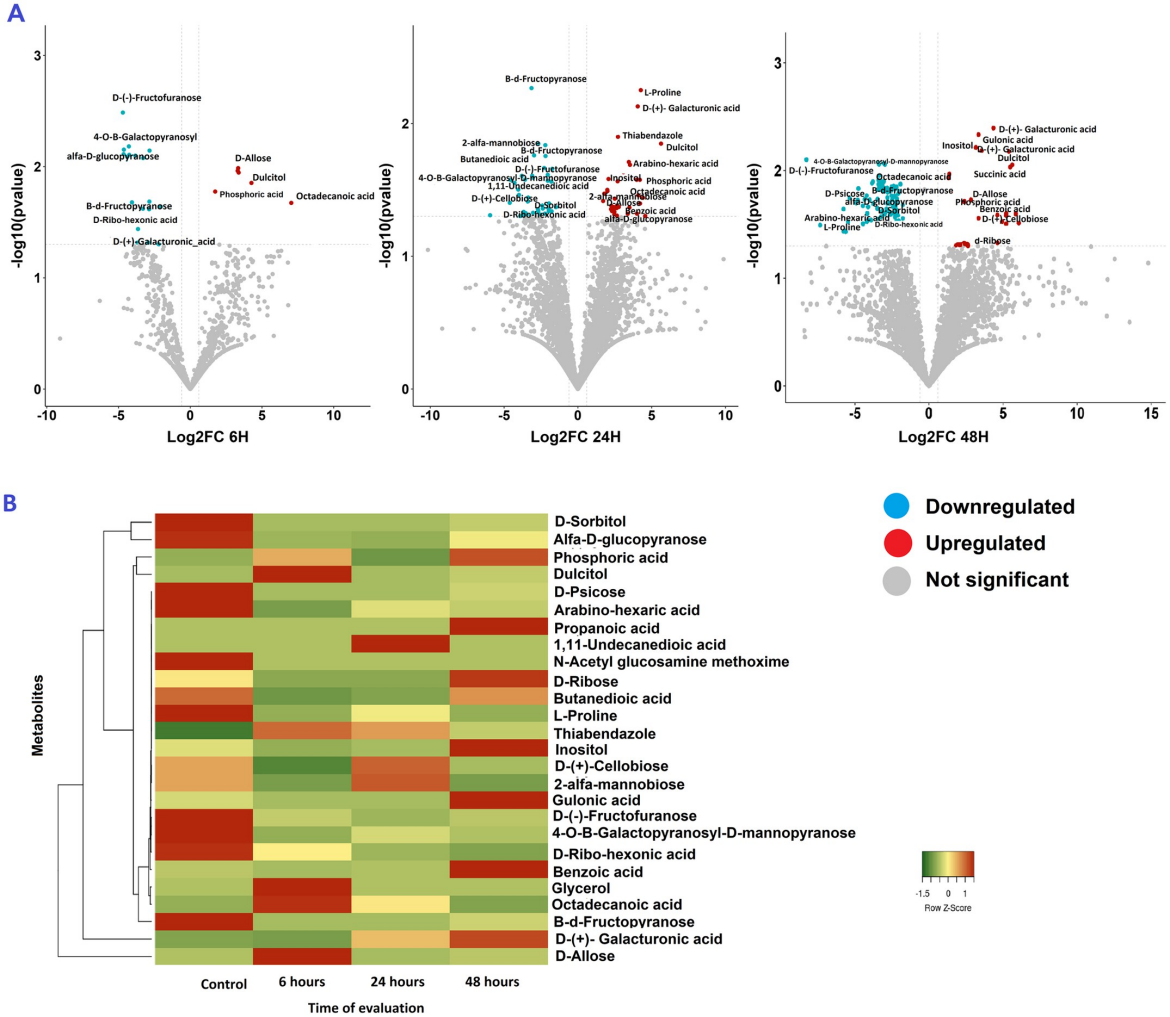
Effect of fungicide exposure on the metabolite profile of resistant *P*. *fijiensis* inoculated with Thiabendazole at different timepoints. (A) Volcano plot of the metabolites obtained at 6, 24 and 48 h of exposure. (B) Heatmap of *P*. *fijiensis* metabolites obtained in GC-MS from strains grown in PDB with 100 μg.mL^-1^ of thiabendazole after 6, 24 and 48 h of exposure.

The volcano plot showed that D-Allose, Dulcitol and Phosphori acid were significantly up-regulated at all the exposure times. Similarly, D-Ribo-hexonic acid, 3-deoxy, D-(-)-Fructofuranose, B-d-Fructopyranose, and 4-O-B-Galactopyranosyl-D-mannopyranos were significantly down-regulated at all exposure times (Fig 3).

**Fig 4:**
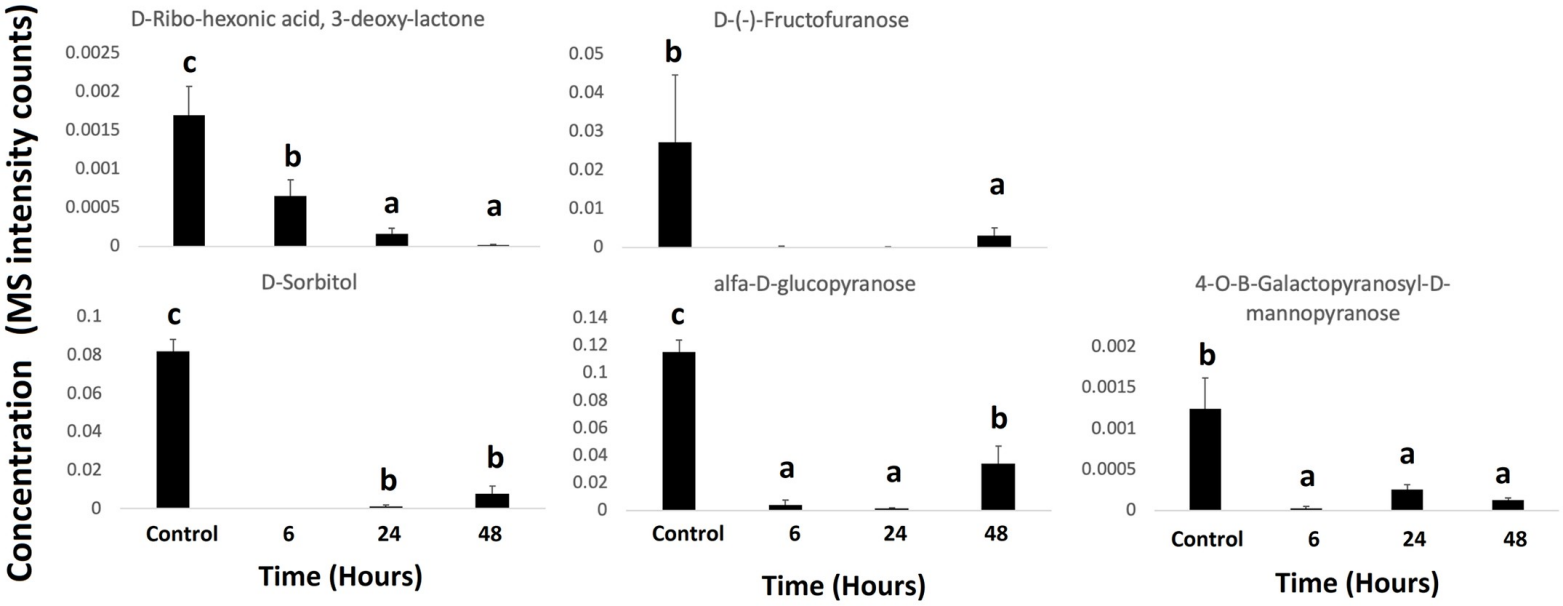
Under-accumulated (down-regulated) metabolites in *P*. *fijiensis* exposed to thiabendazole. Bar charts of metabolites that were significantly under-accumulated in resistant *P*. *fijiensis* isolates exposed to 100 μg.mL^-1^ thiabendazole for 6, 24 and 48 h. Values represent the means and standard errors of 5 replicates. Different letters represent significant differences (p < 0.05).

**Fig 5:**
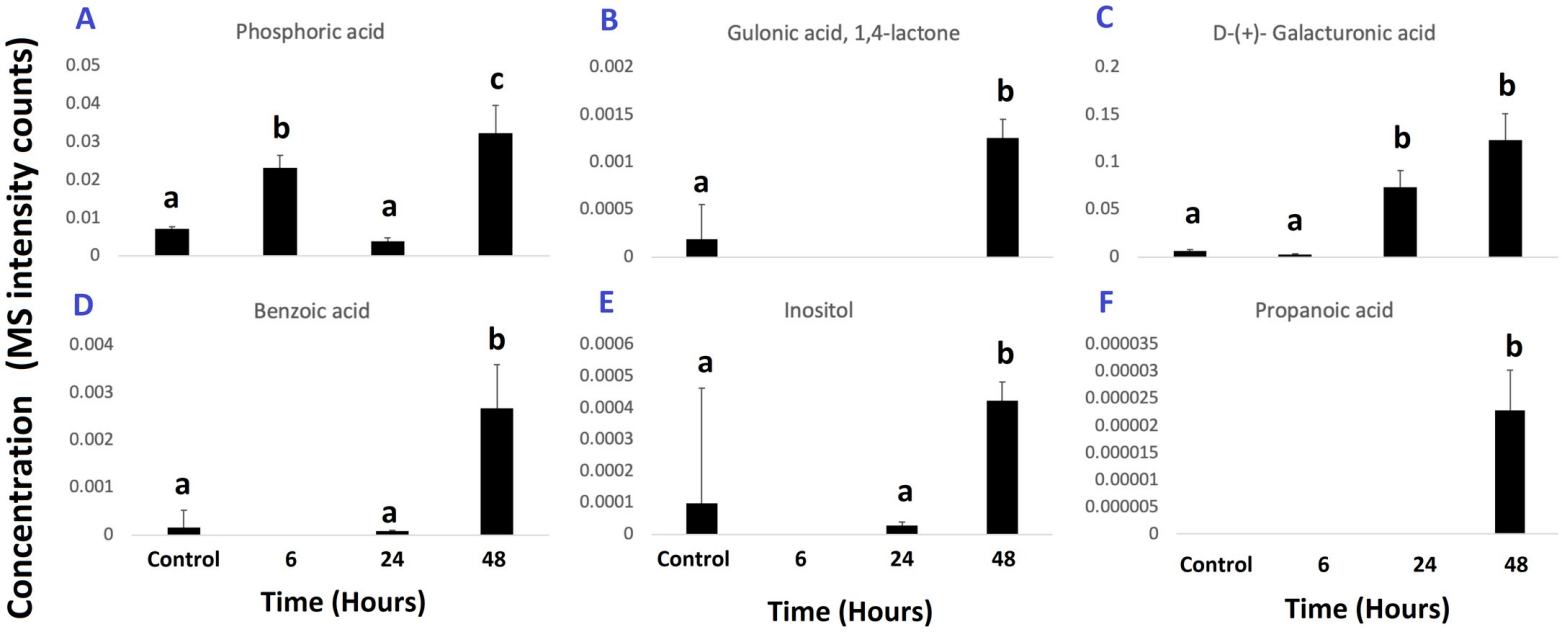
Over-accumulated metabolites in *P*. *fijiensis* exposed to thiabendazole. Bar charts of metabolites that were significantly over-accumulated overtime in resistant *P*. *fijiensis* isolates exposed to 100 μg.mL^-1^ thiabendazole for 6, 24 and 48 h. Values represent the means and standard errors of 5 replicates. Different letters represent significant differences (p < 0.05).

**Fig 6:**
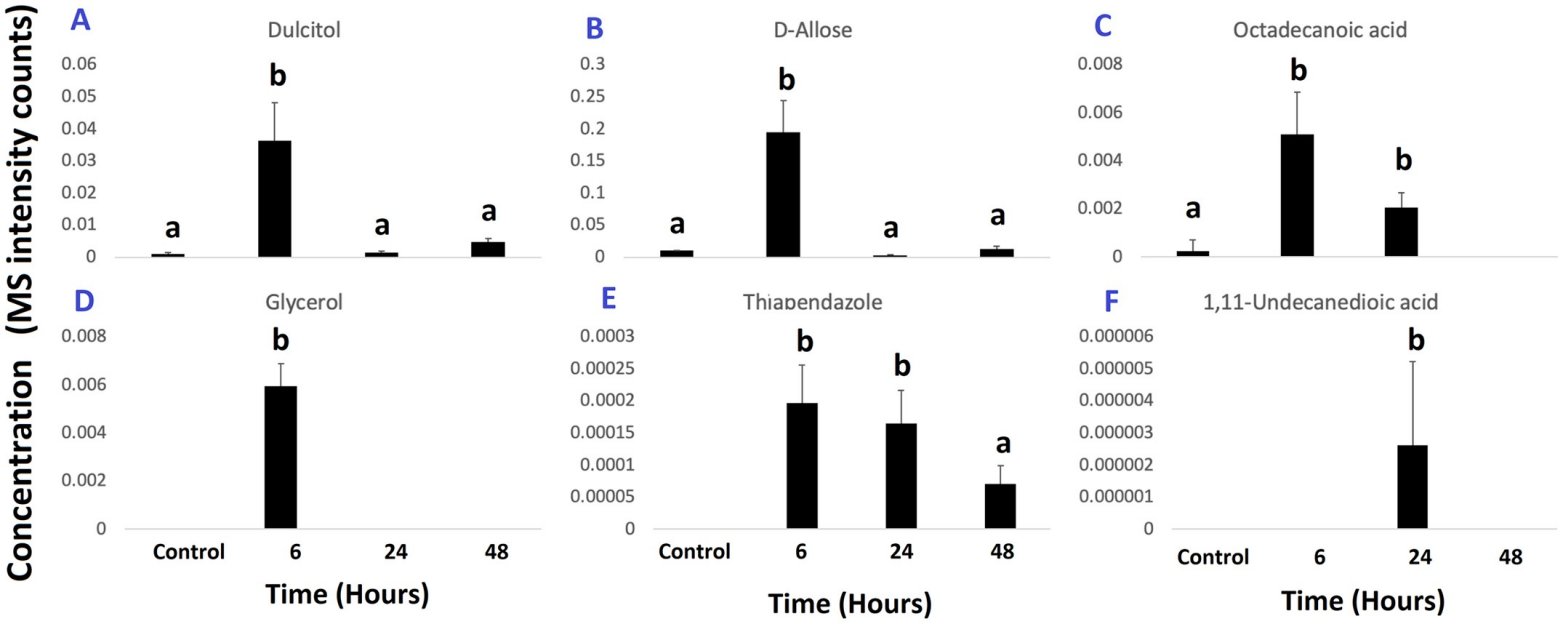
Other differentially-accumulated metabolites in *P*. *fijiensis* exposed to thiabendazole. Bar charts of metabolites of *P*. *fijiensis* that were significantly over-accumulated after exposure to 100 μg.mL^-1^ thiabendazole for 6 or 24 h but showed decreased concentrations at 48 h. Values represent the means and standard errors of 5 replicates. Different letters represent significant differences (p < 0.05).

Notably, compounds such as D-ribo-hexonic acid, 3-deoxy; β-d-fructofuranose, D-sorbitol, β-D-glucopyranose, and 4-O-B-galactopyranosyl-D-mannopyranose, showed highest concentrations in control samples in contrast with the fungicide-exposed samples (Fig 4). However, phosphoric acid, gulonic acid, 1,4 lactone; galacturonic acid, benzoic acid, inositol and propanoic acid showed an increase in concentration during the exposure time with the biggest accumulation shown after 48-h exposure (Fig 5). Similarly, the levels of dulcitol, D-allose, ocatadecanoic acid, glycerol, and 1,11 undecanedioic acid were significantly increased after exposure to the fungicide but decreased thereafter (Fig 6). Interestingly, intracellular thiabendazole was also detected after 6 h exposure, but the concentration decreased afterwards (Fig 6).

## Discussion

To discriminate among thiabendazole resistant and susceptible isolates a sensitivity assay was conducted with six different doses 0, 1, 10, 100, 1,000, and 10,000 μg.mL^-1^. Interestingly, we found different levels of resistance to the fungicide, with isolates that were able to grow at each tested concentration of thiabendazole with MIC values ranging from 10 to 10,000 μg.mL^-1^ (Table 1). This variation in sensitivity does not fit with the expected one-gene single-site interaction previously suggested [6, 10, 17] as single-gene interactions usually show either the presence or absence of the expected response within the population [12]. In contrast, an array of quantitative incremental levels of resistance within the population is commonly associated with multiple gene interactions [12]. Therefore, in this study we aimed to elucidate the impact of thiabendazole on the cell metabolic pathways to provide a better understanding of the possible metabolic adaptations associated with the thiabendazole-resistance mechanisms in *P*. *fijiensis*.

Many compounds related to saturated fatty acids pathways or membrane integrity like inositol, myo-inositol, oleic acid, eicosanoic acid, tetradecanoic acid, tetracosanoic acid, hexadecanoic acid, heptadecanoic acid, 9,12-octadecadienoic acid and octadecanoic acid were produced only or increased their levels in resistant isolates exposed to 10 μg.mL^-1^ of the fungicide. Mannonic acid—a fatty acid precursor and energy reserve related metabolite—was only present in resistant isolates exposed to 10 and 100 μg.mL^-1^ and could be associated with the modulation of the energy reserves during the stress caused by the fungicide. D-(+)-Trehalose, also an energy related metabolite, was only present in resistant isolates exposed to 10 μg.mL^-1^ of the fungicide.

### Increment of myristic acid in *P*. *fijiensis* suggest a key thiabendazole resistance mechanism

Tetradecanoic (myristic) acid was over-accumulated in *P*. *fijiensis* exposed to 10 μg.mL^-1^ (Table 2). Tetradecanoic acid has been associated with the selective increase of β-tubulin concentration via increment of the levels of the diacylglycerol kinase δ enzyme (DGKδ) in other eukaryotic organisms [33]. Sakai et al., demonstrated that the application of tetradecanoic acid to C2C12 mouse cells increases de levels of DGKδ in 23.2% and the β-tubulin level in 18.5%. DGKδ regulates C2C12 myogenic differentiation associated with the β-tubulin formation and development of C2C12 myotubes [33]. Thiabendazole blocks β-tubulin binding, and the over-accumulation of tetradecanoic acid may suggests that the pathogen activated a β-tubulin compensation mechanism that contributed to the modulation of the resistance in isolates that can stand up to 10 μg.mL^-1^ of the fungicide. Further research is needed to assess the relationship of the β-tubulin levels and associated DNA mutations with the production of myristic acid in *P*. *fijiensis*.

The accumulated metabolites profile of resistant isolates exposed to 100 μg.mL^-1^ of thiabendazole showed a higher number of not detected metabolites (metabolites present in control samples but not in fungicide-exposed samples) when compared to the ones detected in isolates exposed to 10 μg.mL^-1^ (Table 2). Results are in accordance with previous studies suggesting a decrease in the metabolic processes of resistant isolates proportional to the fungicide concentration used. In general, results support previous observations of overall reduced fitness of isolates resistant to fungicides [21].

### Early changes in metabolite profiles of *P*. *fijiensis* after 6, 24 and 48 h of fungicide exposure

The metabolite profile of resistant isolates exposed to 100 μg.mL^-1^ of thiabendazole for 6, 24 and 48 h strongly suggest that different metabolic mechanisms are modulated in response to the stress exerted by the fungicide. As shown in the PCA, all of the resistant isolates exposed to 100 μg.mL^-1^ of the fungicide, clustered together after 6 h of exposure, but only partial and no grouping was observed after 24 and 48 h exposure, respectively (Fig 1). Results suggest that the fungicide exerts a profound metabolic change in resistant isolates during the initial exposure, followed by a normalization in the production of many metabolites associated with the elimination of the fungicide from the cell. The intracellular levels of thiabendazole increased after six hours but then decreased over time, while various ABC-transporters pathways where over-accumulated (Table 3). The fungicides efflux by ABC-transporters has been shown as an important resistance mechanism in many fungi [34, 35]. Further research is needed to determine the specific kind of ABC-transporter associated with the thiabendazole efflux. Similarly, many compounds related with energy pathways like α-D-glucopyranose, D-(+)- cellobiose, β-d-fructofuranose, D-psisose, 4-O-β-galactopyranosyl-D-mannopyranose, and butanedioic acid were depleted during the exposure to the fungicide (Table 3). Results agree with previous reports suggesting that the depletion of energy pathways is closely related to the activation of the ABC-transporters in multidrug resistant isolates [35]. Nonetheless, some energy pathways molecules like dulcitol remained upregulated over time (Table 3).

Thiabendazole mode of action also comprise the inactivation of the fumarate reductase enzyme. This enzyme transforms fumarate to succinate, which a very important molecule for the citric acid cycle, mitochondrial respiration, and ATP production [36, 37]. Our results suggest that the *P*. *fijiensis* isolates able to grow at 100 μg.mL^-1^ overcome the fungicide inhibition of the fumarate reductase by incrementing 1.89-fold the concentrations of butanedioic acid (succinic acid) after 48 h of fungicide application (Table 3). Butanedioic acid is the final product of the fumarate reductase and the overaccumulation of this metabolite prevents the inhibition caused by the fungicide to several pathways in the pathogen.

Other metabolites such as phosphoric acid, D-ribose and D -(+)- galacturonic acid—also related to main energy pathways—were differentially accumulated in resistant isolates (Tables 2 and 3). Results agree with previous reports showing that metabolites such as L-proline, benzoic acid and gluonic acid (vitamin c pathway) increase concentrations over time under stress conditions [38–40]. However, further research is needed to determine the metabolic relationship between the upregulated metabolites arabino-hexaric acid, 3-deoxy-2,4,5-tris and d-ribo-hexitol,3-deoxy, and the fungicide resistance.

Overall, the findings from this research support our hypothesis that *P*. *fijiensis* highly resistant isolates to thiabendazole in fact have developed independent resistance mechanisms. These mechanisms potentially include: i) Mutations in the β-tubulin gene, ii) not-yet-described mechanisms involving the increase in the β-tubulin concentrations via overexpression of the diacylglycerol kinase δ enzyme, iii) not-yet-described modulation of the fumarate reductase enzyme to maintain or increase the production of succinic acid over time, and iv) the possible efflux of the fungicide out of the cell via ABC-transporters (Figs 3 and 6, Tables 2 and 3). Mutation in the β-tubulin gene consists of a nucleotide change at codon 198 (GAG instead of GCG) that confers resistance and is the only known resistance mechanism reported in the species for this fungicide [6, 41, 42].

Molecular markers are essential tools to understand fungicide resistant mechanism and cell interactions. However, not all interactions can be described by genetic analysis only. Metabolomics plays an important role for describing the impact and interaction of a specific fungicide on the cell metabolism. This study shows the importance of the metabolomic profiling to elucidate the possible mechanisms involved in *P*. *fijiensis* resistance to thiabendazole.

## Supporting information

S1 Fig Phylogenetic tree of *P*. *fijiensis* isolates used in this study. Phylogenetic tree includes the isolates from Table 1 and the NCBI’s accessions with the highest similarity.
(TIF)

S2 Fig Principal components analysis of *P*. *fijiensis* isolates exposed to 10 (A) and 100 μg.mL^-1^ (B) of thiabendazole. Sample overlap shows that no grouping is observed.
(TIF)
