# Supplementary figures and images for: GC-MS metabolite profiling of Pseudocercospora fijiensis isolates resistant to thiabendazole
Source: PLoS One. 2024 Nov 21;19(11):e0313915. doi: 10.1371/journal.pone.0313915 (PMC11581298; doi:10.1371/journal.pone.0313915)

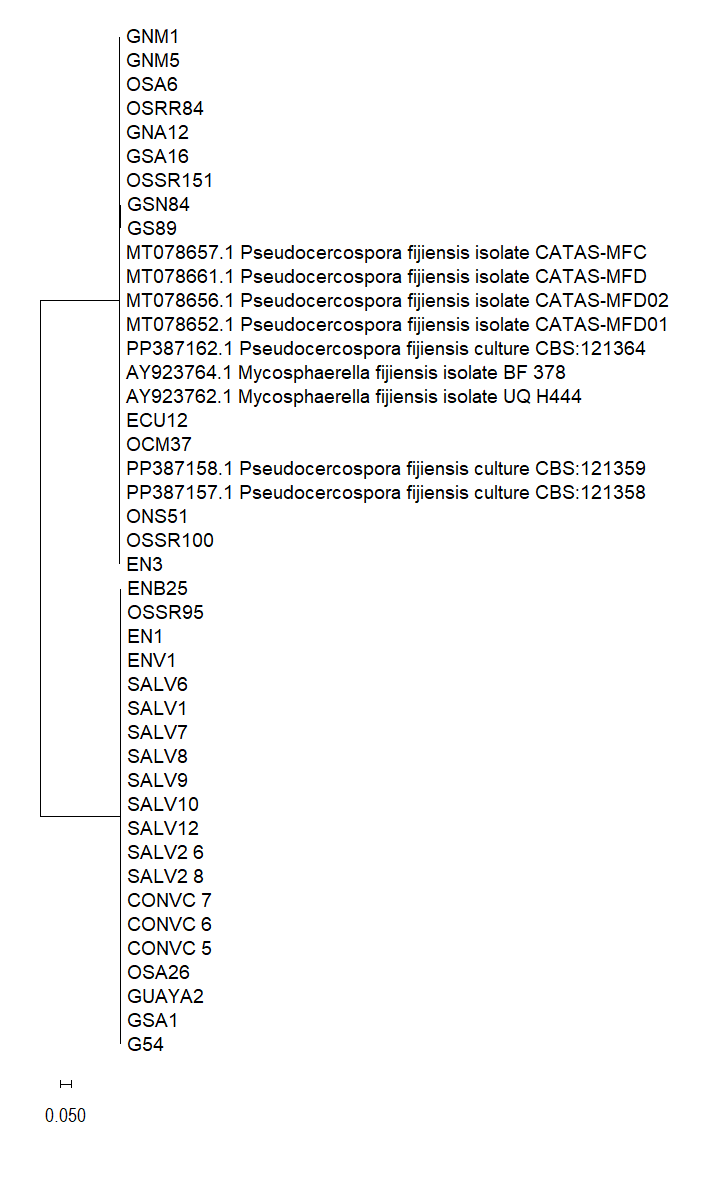

Supplement: S1 Fig — Phylogenetic tree includes the isolates from Table 1 and the NCBI’s accessions with the highest similarity. (TIF) [file pone.0313915.s001.tif]

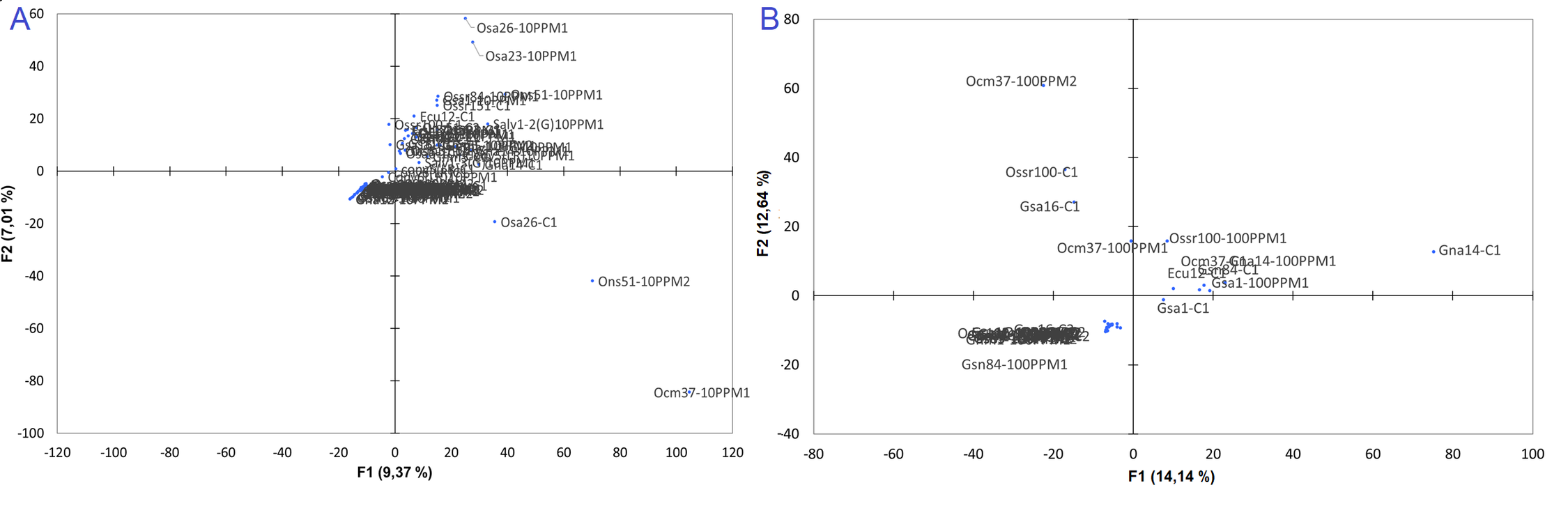

Supplement: S2 Fig — Principal components analysis of P. fijiensis isolates exposed to 10 (A) and 100 μg.mL-1 (B) of thiabendazole. Sample overlap shows that no grouping is observed. (TIF) [file pone.0313915.s002.tif]
